# Informal Caregivers’ Perceptions of Burdens and Benefits Predict Greater Confidence in Their Abilities

**DOI:** 10.1093/geroni/igab046.2938

**Published:** 2021-12-17

**Authors:** Cristina de Rosa, Rebecca Lorenz, Suzanne Sullivan

**Affiliations:** 1 University at Buffalo, Buffalo, New York, United States; 2 University at Buffalo, University at Buffalo, New York, United States

## Abstract

Informal caregivers experience both burdens and benefits from caregiving. This analysis aimed to determine whether caregiver perceptions of burdens and benefits predicted feelings of confidence in their abilities. In the National Study of Caregiving (NSOC) Round II (2015), we identified 1,390 caregivers as “primary” for providing the greatest number of care hours in the past month to individuals age 65 and over. Logistic regression was performed to assess the influence of primary caregivers’ gender, age, relationship to their care recipients, and self-reported indications of burdens and benefits on the odds that they would report confidence in their abilities. Caregivers were more likely to report confidence in their abilities when caregiving taught them to deal with difficult situations (OR=5.93, 95% CI [4.67, 7.54]), gave them satisfaction that their care recipient was well cared for (OR=1.97, 95% CI [1.26, 3.04]), and when caregiving brought them closer to their care recipient (OR=2.61, 95% CI [2.02, 3.36]). Caregivers were less likely to feel confident if they reported frequent changes in routine (OR=.78, 95% CI [.64, .96]). The final model predicted confidence (chi-square = 525.383 [4] p < .001) and correctly classified 78.7% of cases. All other variables were non-significant. These findings suggest that confidence in abilities is influenced by caregivers’ perception of learning to handle difficult situations, satisfaction, closeness to the recipient, and burdens associated with changes in routine. Future research should further explore burdens and benefits of caregiving. Health care providers should routinely assess caregivers and provide referrals for additional resources.

